# Influence of Chicken Manure Fertilization on Antibiotic-Resistant Bacteria in Soil and the Endophytic Bacteria of Pakchoi

**DOI:** 10.3390/ijerph13070662

**Published:** 2016-06-30

**Authors:** Qingxiang Yang, Hao Zhang, Yuhui Guo, Tiantian Tian

**Affiliations:** 1 School of Environment, Henan Normal University, Xinxiang 453007, China; kele1564@126.com; 2 College of Life Sciences, Henan Normal University, Xinxiang 453007, China; gyh@126.com (Y.G.); hsdtiantiantian@126.com (T.T.); 3 Key Laboratory for Microorganisms and Functional Molecules, University of Henan Province, Xinxiang 453007, China

**Keywords:** antibiotic-resistance bacteria, chicken manure, multiple antibiotic-resistant endophytic bacteria, pakchoi, soil, pot experiment

## Abstract

Animal manure is commonly used as fertilizer for agricultural crops worldwide, even though it is believed to contribute to the spread of antibiotic resistance from animal intestines to the soil environment. However, it is unclear whether and how there is any impact of manure fertilization on populations and community structure of antibiotic-resistant endophytic bacteria (AREB) in plant tissues. To investigate the effect of manure and organic fertilizer on endophytic bacterial communities, pot experiments were performed with pakchoi grown with the following treatments: (1) non-treated; (2) chicken manure-treated and (3) organic fertilizer-treated. Manure or organic fertilizer significantly increased the abundances of total cultivable endophytic bacteria (TCEB) and AREB in pakchoi, and the effect of chicken manure was greater than that of organic fertilizer. Further, 16S rDNA sequencing and the phylogenetic analysis indicated that chicken manure or organic fertilizer application increased the populations of multiple antibiotic-resistant bacteria (MARB) in soil and multiple antibiotic-resistant endophytic bacteria (MAREB) in pakchoi. The identical multiple antibiotic-resistant bacterial populations detected in chicken manure, manure- or organic fertilizer-amended soil and the vegetable endophytic system were *Brevundimonas diminuta*, *Brachybacterium* sp. and *Bordetella* sp., suggesting that MARB from manure could enter and colonize the vegetable tissues through manure fertilization. The fact that some human pathogens with multiple antibiotic resistance were detected in harvested vegetables after growing in manure-amended soil demonstrated a potential threat to human health.

## 1. Introduction

Antibiotics were the greatest discovery for improving human health in the 20th century. At present, veterinary antibiotics (VAs) are also commonly incorporated into animal feeds to improve growth rate and feed efficiency in some countries [1]. China is the largest producer and user of antibiotics in the world, based on market sales data [2,3]. In 2013, the total antibiotic usage in China was approximately 162,000 tons, with animal consumption accounting for about 52% of the total antibiotics, and the rest used by humans [4]. It is noteworthy that a high proportion of VAs are mainly excreted via urine and feces, and 30%−90% of them in the feces are parent compounds or metabolites [5]. Subsequently, these residual antibiotics can enter the soil environment following the land application of animal manure at a level of 15,000−150,000 kg·ha^−1^ per year in vegetable farming in China [6].

Animal manure is an important reservoir of antibiotic residues, antibiotic-resistant bacteria (ARB, including human pathogenic bacteria (HPB)) and antibiotic resistance genes (ARGs) [7,8]. Many studies have assessed the persistence, transfer and transformation of residual VAs in soil environments and found that manure fertilization could lead to a significant increase in soil antibiotic detection levels [9,10,11]. Further reports have confirmed the promotion effects of manure application on the prevalence of antibiotic resistance and variation of microbial community structure in soil environments [12,13,14]. Additionally, many reports have shown a positive correlation between the abundance of ARGs and anthropogenic manure application in manure-amended soil [15,16,17]. Our previous research showed a high prevalence of antibiotic-resistant endophytic bacteria (AREB), including some resistant to more than three different types of antibiotics, in various manure-fertilized vegetables including celery, pakchoi and cucumber [5]. The selective pressure from various residual antibiotics and horizontal gene transfer (HGT) are considered to play key roles in the production and spread of environmental antibiotic resistance [18]. However, it is unclear whether ARB themselves, especially some multiple antibiotic-resistant bacteria (MARB), can be directly transferred from manure to soil and even to plants. Few reports have considered the persistence and survival of ARB or MARB from manure in soil environments and their further transfer to plant endophytic systems. Our previous study on field vegetables indicated that there was some relationship between ARB in vegetable endophytic systems and those in chicken or swine manure, and some ARB genera were frequently detected in plant, soil and manure environments [19].

In the present study, a series of pot experiments were established to investigate the influence of manure application on the abundance and diversity of ARB in soil and AREB in pakchoi, and to evaluate whether and how any relationship occurred between ARB from chicken manure, manure-amended soil and manure-fertilized vegetables.

## 2. Materials and Methods

### 2.1. Chemical Reagents

Three technical-grade antibiotics were purchased from Dr. Ehrenstorfer GmbH (Germany) including cephalexin (97.0%), tetracycline (97.0%) and ciprofloxacin (95.0%), corresponding to three classes of antibiotics: β-lactams, tetracyclines and fluoroquinolones, respectively. Stock solutions of the three antibiotics (5 mg·mL^−1^) were prepared by dissolving each compound in distilled water and storing it in a brown bottle at 4 °C. Working solutions were freshly prepared by filtering the stock solutions with a 0.2 µm filter on the day of use.

### 2.2. Samples

Chicken manure (CM, simply piled up for about one month in chicken farms) was collected from three large-scale chicken farms (housing 10,000–15,000 chickens) in the suburb of Xinxiang City, in which the chickens were fed with antibiotics, especially cephalexin, tetracycline and ciprofloxacin, for general disease prevention or treatment. Manure samples from the three farms were mixed as one mixture sample and used in this study. Commercial organic fertilizer (COF) was purchased from local fertilizer providers named “Xiang Rui He Xie”, which was mainly made from chicken feces and occupied more than an 80% share of the market due to its high quality and low price. Soil samples were collected from a nearby forest field without manure fertilization. Each sample was a mixture from at least five locations and immediately stored on ice and transported to the laboratory where the samples were stored in a refrigerator (4 °C) and processed within 48 h. Under sterile conditions, 10 g of fresh samples were put into a flask with 100 mL of sterile water and glass beads and shaken at 200 rpm for 1 h. The suspensions were used for microbial analysis. Pakchoi were harvested from pot tests. Five samples of the vegetables were collected from each pot. Then, the vegetable samples were stored in freshness protection packages at 4 °C and processed within 48 h.

### 2.3. Pot Experimental Procedure

Each of nine plastic pots (700 mm diameter × 170 mm height) was filled with 36 kg of the above dry soil or soil with CM or soil with COF. The nine pots comprised three treatments with three replicates per treatment: (1) control without CM or COF; (2) CM-treated (6 kg of CM and 30 kg of soil); and (3) COF-treated (6 kg of COF and 30 kg of soil).

Of pakchoi (*Brassica chinensis* L.) seeds, 5 g was planted in each pot and irrigated twice a week with deionized water. All pots were incubated in a greenhouse, controlled at 25 ± 2 °C in daylight and 20 ± 2 °C in dark (70% relative humidity). Pakchoi were harvested after 45 d of cultivation for AREB enumeration and multiple antibiotic-resistant endophytic bacteria (MAREB) isolation.

### 2.4. Enumeration of Total Cultivable Heterotrophic Bacteria (TCB), ARB and MARB

Colony-forming units (CFU) of TCB, ARB and MARB were determined by a modified plate dilution technique on meat-peptone agar [20]. The suspensions of soil samples were diluted mostly to 10^−6^ degrees and then an appropriately diluted sample was selected to spread on the plates. The incubation was at 28 °C for 3 d. Three types of antibiotics, cephalexin, tetracycline and ciprofloxacin, were selected for ARB and MARB research in this study for their wide application in these three chicken farms based on our practical investigation. Enumeration of bacteria resistant to cephalexin, tetracycline and ciprofloxacin was conducted under the same conditions on meat-peptone agar supplemented with antibiotics at a final concentration of 100, 16 and 4 μg·mL^−1^, respectively, according to the breakpoint values defined by the Clinical and Laboratory Standards Institute [21]. Enumeration of MARB was determined by mixing three of the above antibiotics at each antibiotic’s final concentration in meat-peptone agar [22]. The numbers of TCB, ARB and MARB were determined by the average of the three replicate samples from each pot.

### 2.5. Enumeration of Total Cultivable Endophytic Bacteria (TCEB), AREB and MAREB

For isolation of endophytic bacteria, fresh vegetables were cleaned with water, air-dried, immersed in 20% hydrogen peroxide for 30 min, followed by rinsing in sterile Milli-Q filtered water (3 min × 3 times). Then, they were immersed in 70% ethanol for 1 min and rinsed as above. Finally, surface-sterilized samples were dried using sterilized filter papers [23]. To ensure the complete surface disinfection, 0.2 mL of the last wash water was spread on meat-peptone agar and cultivated at 30 °C for 3 d to check for colony growth [24]. Of disinfected vegetables, 3 g was cut into pieces and ground together with quartz sand in a sterile mortar. The ground tissue was mixed with 10 mL of sterile water. Each 100 μL of suspension was spread on meat-peptone agar and on various antibiotic-containing agars (cephalexin, tetracycline and ciprofloxacin concentrations were 100, 16 and 4 μg·mL^−1^, respectively) for cultivation at 28 °C for 3 d. Each pot was replicated three times. The CFU of TCEB, AREB and MAREB were enumerated [25].

### 2.6. Isolation and Identification of MARB and MAREB

For MARB and MAREB analysis, each 50 colonies of bacteria growing on antibiotic-containing meat-peptone agars from the three pot experimental treatments were selected and purified according to the different morphological characteristics. Then the genomic DNA of bacterial strain was extracted using CTAB method [26]. The 16S rRNA gene of bacteria was amplified using the universal primer pair, 27F (5′-AGAGTTTGATCCTGGCTCAG-3′) and 1492R (5′-GGTTACCTTGTTACGACTT-3′) [27]. Analysis of 16S rDNA sequences was performed using the NCBI database and nucleotide BLAST (http://blast.ncbi.nlm.nih.gov/Blast.cgi). Sequences were identified as the most closely related species with the highest similarity. A phylogenetic tree was constructed using the neighbor-joining method in MEGA version 4.1 using 1000 bootstrap replications (MEGA, Phoenix, AZ, USA).

### 2.7. Statistical Analysis

A one-way ANOVA was performed to determine significant differences (*p* < 0.05 and *p* < 0.01) between the prevalence of ARB in different samples. Statistical analysis was performed using the software SPSS 21.0 (IBM, Chicago, IL, USA) [28].

## 3. Results and Discussion

### 3.1. Influence of Manure Fertilization on the Prevalence of ARB in Soil

To determine whether CM or COF application influenced the prevalence of cultivable ARB and MARB in soil, plate counts were conducted before and after vegetable planting (Table 1). According to the data shown in Table 1, after being added to CM and COF, the soil-cultivable ARB resistant to cephalexin, tetracycline, ciprofloxacin and MARB resistant to the three antibiotics reached 2.57 × 10^8^ to 3.12 × 10^8^, 1.21 × 10^8^ to 4.63 × 10^8^, 0.79 × 10^8^ to 1.26 × 10^8^ and 0.52 × 10^7^ to 1.01 × 10^7^ CFU·g^−1^, respectively, which was two to three orders of magnitude higher than those in the control pots (1.00 × 10^6^ to 4.00 × 10^6^ CFU·g^−1^ of ARB and 3.00 × 10^4^ CFU·g^−1^ of MARB).

After the vegetable harvest, the concentrations of ARB and MARB in the treated soil markedly decreased by two orders of magnitude compared to before planting (CM-amended soil, from 4.63 × 10^8^ to 4.99 × 10^6^ CFU·g^−1^ for ARB and 1.01 × 10^7^ to 1.07 × 10^5^ CFU·g^−1^ for MARB; correspondingly for COF-amended soil, from 2.57 × 10^8^ to 4.03 × 10^6^ CFU·g^−1^ and 5.24 × 10^6^ to 6.40 × 10^4^ CFU·g^−1^), while these data showed no significant change in the controls. The results suggested that the sudden increase in ARB and MARB prevalence due to CM or COF application could be dramatically decreased after 45 d of vegetable farming. This decrease was possibly related to the inability to survive in the soil environment for most ARB or MARB from CM or COF, or diffusion and spread of some ARB or MARB from soil to plants. However, it is noteworthy that although the prevalence of ARB or MARB decreased with vegetable farming, their abundances in CM- or COF-amended soils were still significantly higher than those in the controls after harvest (5.00 × 10^6^ to 2.80 × 10^7^ CFU·g^−1^ ARB and 1.10 × 10^5^ CFU·g^−1^ MARB for CM-amended soil and 4.00 × 10^6^ to 1.70 × 10^7^ CFU·g^−1^ ARB and 0.60 × 10^5^ CFU·g^−1^ MARB for COF-amended soil; *p* < 0.05 between CM- or COF-amended soil and control soil samples). This phenomenon implied that there might be a long influence of CM or COF application on micro-ecosystems in agricultural soil. As an example, some VAs, such as tetracyclines, sulfonamides or fluoroquinolones, may persist for several months to years in agricultural fields with manure fertilization [29,30,31]. In soil, these substances may then affect the structure and function of bacterial communities, and the development and spread of ARGs and associated mobile genetic elements [32]. Our previous survey also indicated a higher prevalence of ARB in a CM-amended field than manure-free soils [5]. The input of antibiotic-resistant bacteria from manure to soil, the enhancement of soil indigenous resistant bacteria under the pressure of residue antibiotics from manure, as well as the horizontal gene transfer would result in such an increase of ARB and MARB. Xu et al. reported that the counts of ciprofloxacin-resistant bacteria in soil were distinctly increased with the application of swine manure, and significant correlations were observed between (fluoro)quinolone resistance levels and swine manure application rates [11]. However, our results showed that the abundances of ARB and MARB in COF-amended soil were lower than those in CM-amended soil. Compared with CM, COF had significantly reduced concentrations of antibiotic residues and population counts of ARB from raw material due to the special processing that includes elevated temperature, desiccation, molding and extrusion [33]. In this study, only three types of antibiotics, cephalexin, tetracycline and ciprofloxacin, were used to research ARB and MARB due to their most frequent applications in the sampled chicken farms. As in our previous investigation, other antibiotics such as sulphonamides, aminoglycosides, etc., were also widely used in chicken or swine farms, which should be studied in the future [19].

### 3.2. Influence of Manure Fertilization on the Prevalence of AREB in Pakchoi

To clarify whether ARB in the CM- or COF-amended soil were retained on or in the vegetables, the abundances of TCEB, AREB (including endophytic bacteria resistant to cephalexin, tetracycline and ciprofloxacin) and MAREB resistant to the three antibiotics in pakchoi tissues were determined by viable plate count (Table 2).

Compared to controls, various AREB and MAREB were detected at one to two orders of magnitude greater in CM- or COF-amended pakchoi samples. Among the tested antibiotics, AREB resistant to cephalexin was the most prevalent in the investigated samples, even in controls, indicating that a high level of indigenous cephalexin-resistant bacteria existed in the pakchoi endophytic system. This is likely related to the high levels of cephalexin-resistant bacteria in the control and treated soils (Table 1). This result coincided with our field research, in which the most prevalent AREB was also cephalexin-resistant bacteria in greenhouse pakchoi and celery [5]. It was notable that a high proportion of MAREB was generally detected in the pakchoi endophytic bacteria—especially in the CM-treated samples, with close to 1%. The tested antibiotics in this study are important human prescription drugs. Our survey indicated that they are widely applied in the nearby chicken farms and generally at drug dosages exceeding the recommended limit [5]. High prevalence of ARB and MARB in chicken manure, COF and CM-/COF-amended soils led to the high detection rates of AREB and MAREB in pakchoi. However, whether these AREB and MAREB can be transferred to humans through food and further threaten human health requires a series of further investigations.

### 3.3. Phylogenetic Relationship of MARB and MAREB in CM- or COF-Fertilized Soil and Endophytic System of Pakchoi

A total of 25 genera of MARB and MAREB were isolated and identified, based on the full length of the 16S rDNA sequences from the three experimental pot treatments, among which 10 genera were isolated from controls. Another 14 and 19 genera were isolated from CM- and COF-amended treatments, respectively. Dominant genera of MARB from soil before planting and MAREB from vegetables at harvest in different treatments are presented in Table 3. Figures S1–S3 (Supplementary Materials) show the detailed phylogenetic compositions of MARB and MAREB.

In controls, two genera of MARB (*Stenotrophomonas* and *Brevundimonas*) were obtained from the soil samples before harvest, and two genera of MAREB (*Stenotrophomonas* and *Microbacterium*) were obtained from the vegetable samples. *Stenotrophomonas maltophilia* was found in both the soil and vegetable endophytic system, occupying 60.0% and 77.78% of the total MAR(E)B in the control soil and pakchoi, respectively. According to reports, *S. maltophilia* is one of the most prevalent opportunistic Gram-negative bacilli, which is increasingly recognized as an important nosocomial pathogen causing infections in debilitated or immune-suppressed patients [34]. In the environment, most isolates of this species are resistant to multiple antibiotics including tetracyclines, fluoroquinolones, chloramphenicol, macrolides, aminoglycosides, polymyxins, cephalosporins and β-lactam antibiotics due to their low membrane permeability, expression of antibiotic-modifying enzymes and efflux pumps [35]. *S. maltophilia* has also been shown to acquire genes involved in antibiotic resistance from Gram-positive bacteria and to transfer antibiotic resistance to other bacteria [36,37,38]. Our results indicated that this bacteria was an indigenous MARB and freely moved between soil and plants.

Four major genera were isolated from the CM samples: *Brevundimonas*, *Brachybacterium*, *Enterococcus* and *Zimmermannella*. Simultaneously, genus *Brachybacterium* was isolated from CM-amended soil and vegetable samples with high percentages. As a kind of familiar microbial population in environments, *Brachybacterium* widely exist in soil, marine sediment and plant roots [39,40,41,42]. For example, *Brachybacterium saurashtrense* was isolated from the roots of halophyte *Salicornia brachiata* as halotolerant diazotrophic bacteria, and showed significant plant growth-promoting activities in *Salicornia* in salt stress conditions [39]. *Brachybacterium phenoliresistens*, which was isolated both in CM and the vegetable endophytic system in the present study, has been reported to degrade hydrocarbons under high salinity conditions [40]. Another bacteria, *Brevundimonas diminuta*, was also isolated from CM and vegetable samples. This species is an environmental Gram-negative bacillus that is intrinsically resistant to fluoroquinolones, and some species can cause human infections involving the bloodstream, intravascular catheters, the urinary tract and the pleural space [43]. Therefore, their distribution in the environments and the risks to human health are worthy of concern. In this study, the consistency of MARB in CM, vegetable and CM-amended soil suggested that MARB from CM could enter and colonize the vegetable tissues through CM fertilization.

There were 14 genera of MARB detected in the COF-amended soil samples: *Brachybacterium*, *Promicromonospora*, *Microbacterium*, *Luteimonas*, *Sphingomonas*, *Bosea*, *Paenibacillus*, *Dietzia*, *Sphingobacterium*, *Chitinophaga*, *Isoptericola*, *Sphingopyxis*, *Bordetella* and *Pseudomonas*. Although the levels of antibiotic resistance in COF-amended soils were not as high as in CM-amended soils, the diversity of obtained MARB was much higher in this study, though the reason remains unknown. We speculate that COF was collected from different chicken farms which used different antibiotics, while the CM was collected from only one farm. However, from the COF-amended pakchoi, only two genera of MAREB bacteria (seven strains), *Brachybacterium* and *Bordetella*, were isolated and identified. Similar to the CM-amended pakchoi, *Brachybacterium* was isolated from the endophytic system. *Bordetella* is a genus of small (0.2–0.7 µm) Gram-negative coccobacilli of the phylum Proteobacteria. Three species of *Bordetella* are human pathogens: *B. pertussis*, *B. parapertussis* and *B. bronchiseptica*. Fang et al. reported that *B. pertussis* was one of the dominant HPB in manures [6]. It was the causative agent of whooping cough and continued to circulate among children and adolescents even in regions with high vaccine coverage [44]. It is notable that Bordetella was isolated from both COF-amended soil and the vegetable endophytic system.

Although the obtained MAREB fell into very limited genera (*Stenotrophomonas* and *Microbacterium* from the control samples; *Brevundimonas* and *Brachybacterium* from CM-amended vegetables; and *Brachybacterium* and *Bordetella* from COF-amended vegetables), they were obviously related to their own soil environmental bacterial populations. In our previous study on field antibiotic resistance, the possibility of transfer of ARB from livestock manures to soils and the persistence of ARB in these environments were proposed [19]. The present study further confirmed this relationship and suggested the possibility of further transfer of MARB from CM- or COF-amended soil to the plant endophytic system.

## 4. Conclusions

In this study, pakchoi was grown in pot experiments to investigate whether and how the application of CM or COF affected soil and plant endophytic AR(E)B or MAR(E)B. The application of CM or COF significantly increased the prevalence of ARB in soil and AREB in pakchoi, with COF much weaker than CM. Such influence on the soil microbial community weakened with pakchoi growth but was still detectable after 45 d. Comparison of MARB and MAREB isolates among CM, CM-/COF-amended soil and vegetables showed that most types of MARB could not enter into the plant endophytic system through manure fertilization. However, three types of MAREB, *Brevundimonas diminuta*, *Brachybacterium* sp. and *Bordetella* sp., were simultaneously present in CM- or COF-amended soil and vegetable endophytic bacterial systems but absent in controls, indicating their possible transfer from CM or COF to plants along with the fertilization process.

## Figures and Tables

**Table 1:** Concentrations and rates of ARB and MARB in different treatment soils before and after growing pakchoi (CFU·g^−1^).

Samples		TCB (×10^9^)	Cep^r^		Tet^r^		Cip^r^		MARB	
			ARB (×10^8^)	Rates (%)	ARB (×10^8^)	Rates (%)	ARB (×10^8^)	Rates (%)	(×10^6^)	Rates (%)
CM		2.33 ± 0.38	15.01 ± 1.94	64.55	6.80 ± 0.90	29.18	4.26 ± 0.55	18.30	60.60 ± 0.07	2.60
COF		3.47 ± 0.13	7.15 ± 2.08	20.63	2.57 ± 0.82	7.42	0.59 ± 0.25	1.70	2.57 ± 0.04	0.07
Control	before	0.02 ± 0.11	0.04 ± 2.05	20.54	0.01 ± 0.57	6.70	0.01 ± 0.40	3.95	0.03 ± 0.01	0.14
	after	0.03 ± 0.43	0.06 ± 0.64	19.67	0.01 ± 0.14	5.27	0.01 ± 0.43	2.96	0.02 ± 0.01	0.06
CM-amended	before	1.66 ± 0.97	3.12 ± 1.80	34.09	4.63 ± 0.87	28.18	1.26 ± 0.35	5.72	10.10 ± 0.03	0.61
	after	0.44 ± 0.34	0.28 ± 0.81	6.76	0.09 ± 0.31	2.13	0.05 ± 0.06	1.16	0.11 ± 0.01	0.02
COF-amended	before	1.13 ± 0.87	2.57 ± 1.13	22.66	1.21 ± 0.53	10.59	0.79 ± 0.31	6.70	5.24 ± 0.01	0.39
	after	0.13 ± 0.53	0.17 ± 0.69	14.01	0.04 ± 0.10	3.51	0.05 ± 0.39	3.90	0.06 ± 0.02	0.05

ARB: antibiotic-resistant bacteria, CM: chicken manure, COF: commercial organic fertilizer, Cep^r^: cephalexin-resistant bacteria, Tet^r^: tetracycline-resistant bacteria, Cip^r^: ciprofloxacin-resistant bacteria, MARB: bacteria resistant to the three antibiotics. The values in the table are average of three samples replicates.

**Table 2:** Concentrations and rates of AREB and MAREB in pot-grown pakchoi (CFU·g^−1^).

Samples	TCEB (×10^4^)	Cep^r^		Tet^r^		Cip^r^		MAREB	
		AREB (×10^3^)	Rates (%)	AREB (×10^2^)	Rates (%)	AREB (×10^2^)	Rates (%)	(×10^2^)	Rates (%)
Control	0.45 ± 0.67	0.29 ± 1.05	6.44	0.98 ± 0.88	2.18	0.53 ± 0.10	1.18	0.17 ± 0.11	0.38
CM-amended	2.44 ± 0.56	3.20 ± 1.44	13.11	8.98 ± 0.29	3.68	3.62 ± 0.25	1.48	2.09 ± 0.12	0.86
COF-amended	2.91 ± 0.70	2.15 ± 1.41	7.39	3.71 ± 0.43	1.27	3.17 ± 0.16	1.09	1.19 ± 0.06	0.41

AREB: antibiotic-resistant endophytic bacteria, CM: chicken manure, COF: commercial organic fertilizer, Cep^r^: cephalexin-resistant endophytic bacteria, Tet^r^: tetracycline-resistant endophytic bacteria, Cip^r^: ciprofloxacin-resistant endophytic bacteria, MAREB: endophytic bacteria resistant to the three antibiotics. The values in the table are average of three samples replicates.

**Table 3:** Dominant genera of MARB and MAREB in CM- or COF-treated soil-pakchoi systems.

Samples		Identification of MAR(E)B	Rates of MAR(E)B (%)
Control (33 strains)	soil	*Stenotrophomonas* (*Stenotrophomonas maltophilia*)	60.00
		*Brevundimonas* (*Brevundimonas* sp.)	40.00
	pakchoi	*Stenotrophomonas* (*Stenotrophomonas maltophilia*)	77.78
		*Microbacterium* (*Microbacterium oxydans*)	22.22
CM-amended (43 strains)	CM	*Brevundimonas* (*Brevundimonas diminuta*)	33.33
		*Brachybacterium* (*Brachybacterium phenoliresistens*)	33.33
		*Enterococcus* (*Enterococcus* *faecalis*)	16.67
		*Zimmermannella* (*Zimmermannella faecalis*)	16.67
	soil	*Microbacterium* (*Microbacterium* *pseudoresisten*; *Microbacterium* *keratanolyticu*; *Microbacterium* sp.)	41.67
		*Isoptericola* (*Isoptericola* sp.)	25.00
		*Brachybacterium* (*Brachybacterium* sp.)	8.33
		*Corynebacterium* (*Corynebacterium* *stationis*)	8.33
		*Promicromonospora* (*Promicromonospora* sp.)	8.33
		*Xanthomonas* (*Xanthomonas bacterium*)	8.33
	pakchoi	*Brachybacterium* (*Brachybacterium phenoliresistens*)	66.67
		*Pseudomonas* (*Pseudomonas* *geniculata*)	22.22
		*Brevundimonas* (*Brevundimonas* *diminuta*)	11.11
COF-amended (50 strains)	soil	*Promicromonospora* (*Promicromonospora* sp.)	21.74
		*Brachybacterium* (*Brachybacterium* sp.; *Brachybacterium* *paraconglomeratum*; *Brachybacterium* *conglomeratum*)	17.39
		*Microbacterium* (*Microbacterium* sp.; *Microbacterium* *binotii*)	8.69
		*Bosea* (*Bosea* *vestrisii*)	4.74
		*Chitinophaga* (*Chitinophaga* sp.)	4.74
		*Bordetella* (*Bordetella* sp.)	4.74
		*Sphingomonas* (*Sphingomonas* *koreensis*)	4.74
		*Luteimonas* (*Luteimonas* sp.)	4.74
		*Paenibacillus* (*Paenibacillus* sp.)	4.74
		*Dietzia* (*Dietzia* sp.)	4.74
		*Sphingobacterium* (*Sphingobacterium arenae*)	4.74
		*Isoptericola* (*Isoptericola variabilis*)	4.74
		*Sphingopyxis* (*Sphingopyxis* sp.)	4.74
		*Pseudomonas* (*Pseudomonas* sp.)	4.74
	pakchoi	*Brachybacterium* (*Brachybacterium* sp.)	83.33
		*Bordetella* (*Bordetella* sp.)	16.67

## Supplementary Materials

The following are available online at www.mdpi.com/1660-4601/13/7/662/s1, Figure S1: Phylogenetic analysis of MARB and MAREB isolated from the control soil and pakchoi; Figure S2: Phylogenetic analysis of MARB and MAREB isolated from the CM-amended soil and pakchoi; Figure S3: Phylogenetic analysis of MARB and MAREB isolated from the COF-amended soil and pakchoi.

## Abbreviations

The following abbreviations are used in this manuscript:

VA	veterinary antibiotic
ARB	antibiotic-resistant bacteria
AREB	antibiotic-resistant endophytic bacteria
MARB	multiple antibiotic-resistant bacteria
MAREB	multiple antibiotic-resistant endophytic bacteria
TCB	total cultivable heterotrophic bacteria
TCEB	total cultivable endophytic bacteria
HPB	human pathogenic bacteria
ARG	antibiotic resistance gene
HGT	horizontal gene transfer
CM	chicken manure
COF	commercial organic fertilizer
CFU	colony-forming units
CTAB	cetyltrimethylammonium bromide
