# Supplementary material for: Influence of Chicken Manure Fertilization on Antibiotic-Resistant Bacteria in Soil and the Endophytic Bacteria of Pakchoi
Source: Int J Environ Res Public Health. 2016 Jun 30;13(7):662. doi: 10.3390/ijerph13070662 (PMC4962203; doi:10.3390/ijerph13070662)
Supplement: Supplementary file 1 [file ijerph-13-00662-s001.pdf]

Supplementary Materials: Influence of Chicken Manure Fertilization on Antibiotic-Resistant Bacteria in Soil and the Endophytic Bacteria of Pakchoi

Qingxiang Yang, Hao Zhang, Yuhui Guo and Tiantian Tian

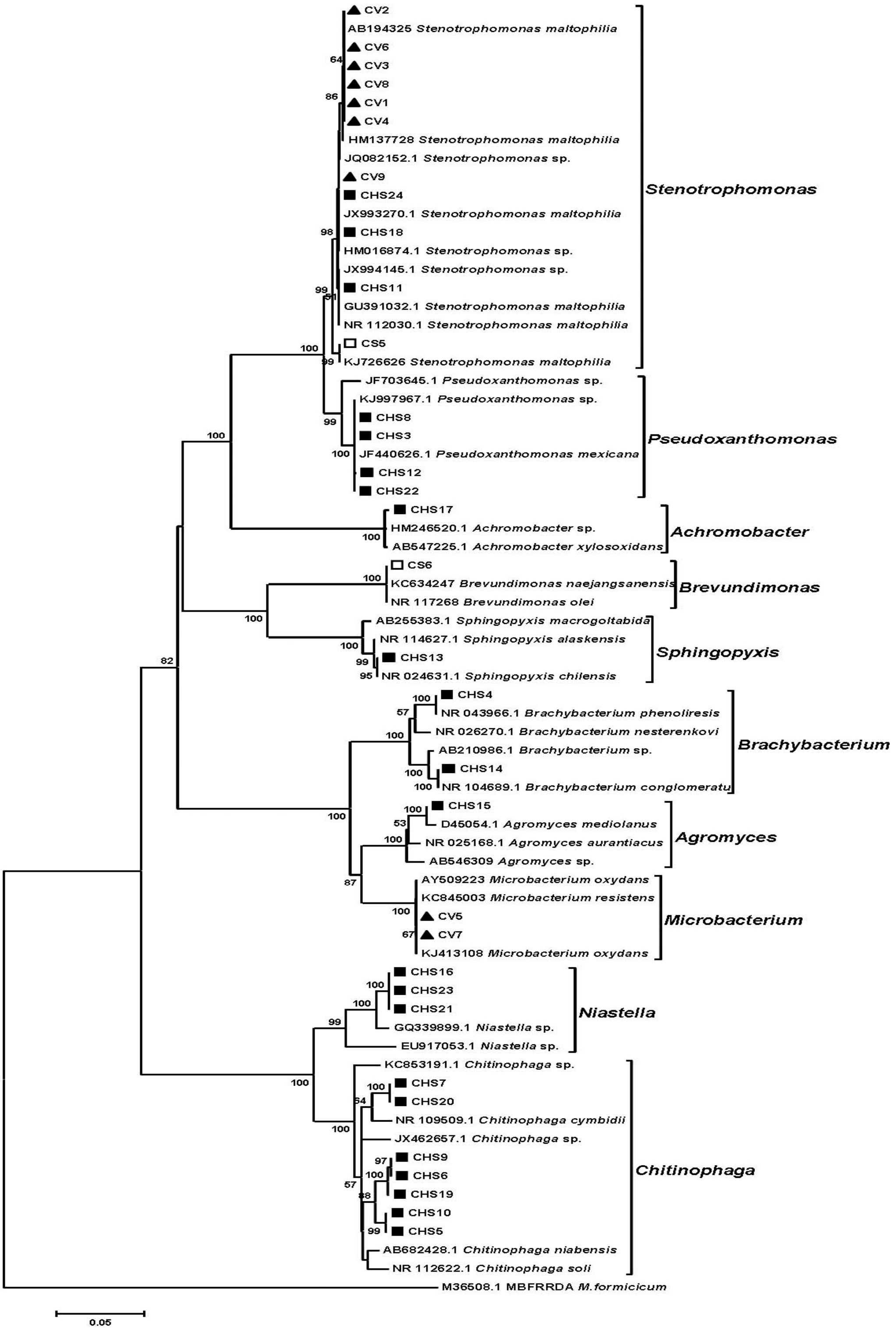

Figure S1. Phylogenetic analysis of MARB and MAREB isolated from the control soil and pakchoi. □, MARB from soil before planting; ▲, MAREB from pakchoi; ■, MARB from soil after harvest.

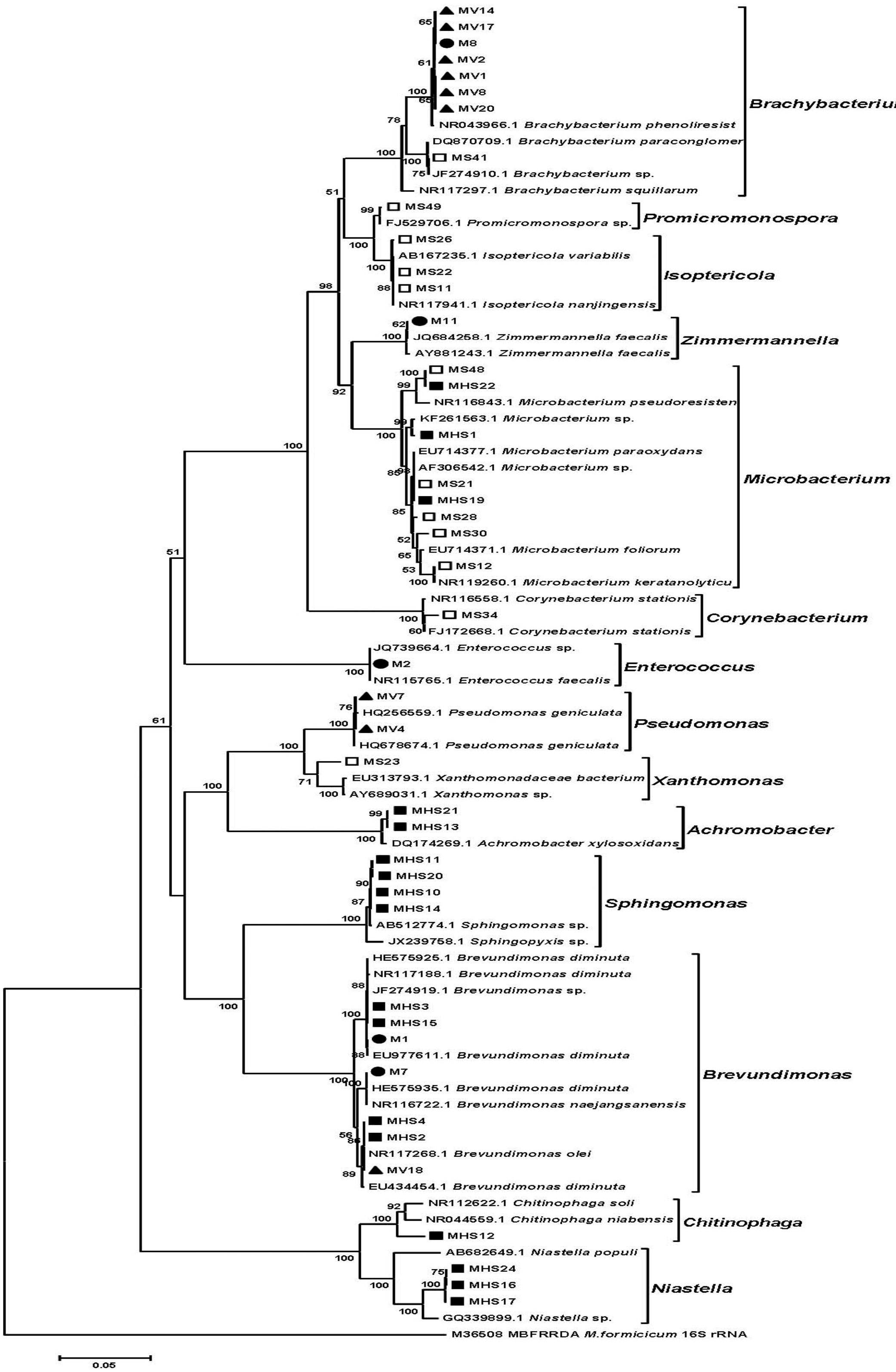

Figure S2. Phylogenetic analysis of MARB and MAREB isolated from the CM-amended soil and pakchoi. □, MARB from soil before planting; ▲, MAREB from pakchoi; ■, MARB from soil after harvest; ●, MARB from CM.

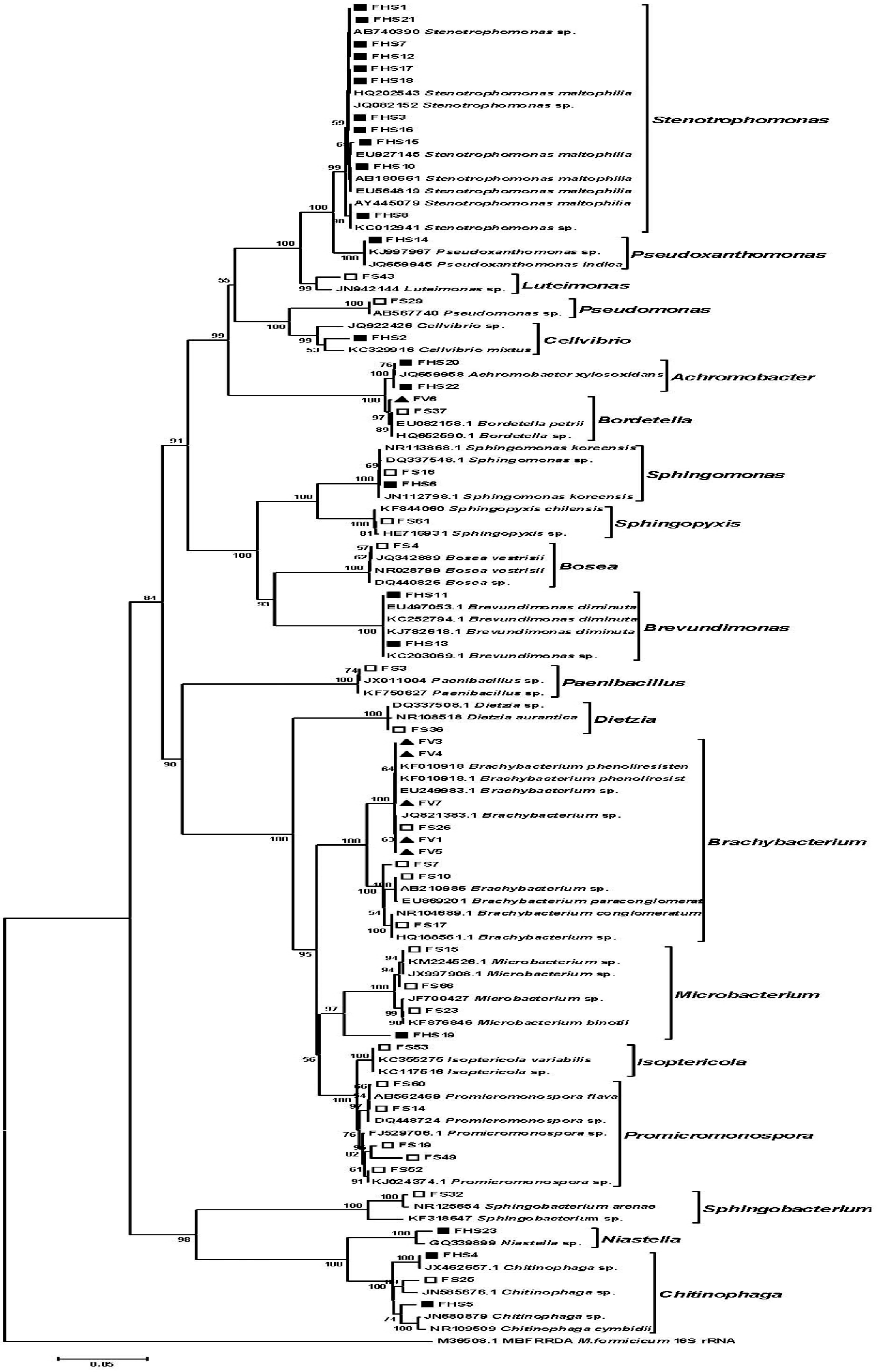

Figure S3. Phylogenetic analysis of MARB and MAREB isolated from the COF-amended soil and pakchoi. □, MARB from soil before planting; ▲, MAREB from pakchoi; ■, MARB from soil after harvest.

© 2016 by the authors; licensee MDPI, Basel, Switzerland. This article is an open access article distributed under the terms and conditions of the Creative Commons by Attribution (CC-BY) license (http://creativecommons.org/licenses/by/4.0/).
